# Effects of the novel pesticide flupyradifurone (Sivanto) on honeybee taste and cognition

**DOI:** 10.1038/s41598-018-23200-0

**Published:** 2018-03-21

**Authors:** Hannah Hesselbach, Ricarda Scheiner

**Affiliations:** University of Würzburg, Biocenter, Behavioral Physiology and Sociobiology, Am Hubland, 97074 Würzburg, Germany

## Abstract

Due to intensive agriculture honeybees are threatened by various pesticides. The use of one group of them, the neonicotinoids, was recently restricted by the European Union. These chemicals bind to the nicotinic acetylcholine receptor (nAchR) in the honeybee brain. Recently, Bayer AG released a new pesticide by the name of “Sivanto” against sucking insects. It is assumed to be harmless for honeybees, although its active ingredient, flupyradifurone, binds nAchR similar to the neonicotinoids. We investigated if this pesticide affects the taste for sugar and cognitive performance in honeybee foragers. These bees are directly exposed to the pesticide while foraging for pollen or nectar. Our results demonstrate that flupyradifurone can reduce taste and appetitive learning performance in honeybees foraging for pollen and nectar, although only the highest concentration had significant effects. Most likely, honeybee foragers will not be exposed to these high concentrations. Therefore, the appropriate use of this pesticide is considered safe for honeybees, at least with respect to the behaviors studied here.

## Introduction

Honeybees (*Apis mellifera*) play an essential role in crop pollination and are thus crucial for human nutrition^1,2^. Depending on their environment, honeybees have to deal with parasites, diseases, habitat loss, pesticides and other stressors^3^. In modern agriculture, pesticides are widely used^4,5^. Particularly the group of neonicotinoids are considered to have negative effects on honeybee longevity and cognition. These chemical substances bind to ion channels within the insect nervous system, particularly in the antennal lobes and the mushroom body^6^. These brain areas are essential for memory formation and integration of visual, tactile and olfactory stimuli^7^. The target of neonicotinoids is the nicotinic acetylcholine receptor (nAchR), where they act agonistically^8^. The AchR plays an important role in tactile and olfactory learning and memory and is thus essential for foraging behavior^6^.

Especially one neonicotinoid, imidacloprid [1-(6-chloro-3-pyridylmethyl)-2nitroimino-imidazolidine], has been examined in detail. Numerous studies show that sublethal doses of imidacloprid not only cause changes in motor behavior^9^, but also impair tactile and olfactory learning and memory^10–13^ and foraging behavior^14^. Cholinergic pesticides generally lead to neuronal inactivation, which could be responsible for cognitive impairments^15^.

The EU decided to restrict the use of the neonicotinoids clothianidin, thiamethoxam and imidacloprid in 2013 because a high risk for honeybees could not be excluded^16^. In 2016 the European Food Safety Authority (EFSA) even went one step further and concluded that some of the previous exceptions also entailed high risks for pollinators^17^.

Recently Bayer AG (Bayer AG, Crop Science Division, Monheim am Rhein, Germany) launched a new pesticide called Sivanto with flupyradifurone (4-[(2,2-difluoroethyl)amino]-2(5 H)-furanone) as active ingredient (ai). This pesticide belongs to Bayer Crop Science’s own chemical class of butenolides^18^. Flupyradifurone was approved in the EU in 2015^19^ but is not yet available on the European market. In the US, it has been in use since 2015. Generally it can be used against sucking pests and especially to control whitefly and aphid species expressing metabolic mechanisms of resistance against neonicotinoid insecticides^18^.

Similar to neonicotinoids, flupyradifurone acts as a reversible agonist on insect nAchR. In contrast to neonicotinoids, however, flupyradifurone acts at a different site of action and thus the structure-activity relationship is different to those formed by neonicotinoids^18^.

Flupyradifurone is toxic for honeybees on an acute oral exposure basis (LD_50_ 1.2 µg ai/bee), whereas it is practically nontoxic to adult bees on an acute contact exposure basis suggesting that ingestion through residues in pollen or nectar is the primary route of concern^18^.

The aim of this study is to investigate effects of flupyradifurone on taste and cognition in honeybee foragers. Normal taste behavior of honeybees is essential for evaluating a nectar or pollen source adequately. Therefore, bees with strongly reduced taste for sugars would be deleterious for a colony, because they would not find an acceptable nectar source. Cognitive tasks such as efficient learning and memory formation are similarly essential for honeybee foragers, because they enable them to relocate floral pollen and nectar resources, efficiently collect nectar and pollen, and navigate accurately when foraging in the field^20^. Because only foragers collect food in a honeybee colony, their successful exploitation of food sources is an important prerequisite for the growth and survival of the entire colony.

To examine the gustatory responsiveness one can test the proboscis extension response (PER) of the honeybees. Gustatory responsiveness is linked to the physiological state of a bee and can also be used to test effects of diseases and pesticides^21^. To test a bee for her learning and memory capability, classical conditioning of the PER is a main learning protocol. Here the bee learns to associate a previously neutral stimulus (conditioned stimulus, CS) with a biologically relevant stimulus (unconditioned stimulus, US). During olfactory conditioning an odor is presented to the bees antennae, followed by an antennal sucrose stimulation that elicits the PER and a reward applied to the proboscis^21^. Though these tests have never been validated by ring-testing such as are used for regulatory risk assessment of pesticides, the PER tests are widely recognized. As they can be done under standardized conditions and are not as elaborate as field and semi-field experiments, they can be used to estimate sublethal effects of pesticides on honeybees. Also comparisons of sensitivity of the bees as well as estimation of no observed effect concentrations, which are useful for regulatory purposes, are possible^22^. Furthermore for imidacloprid Decourtye *et al*. showed that the behavioral toxicity observed in the laboratory at individual level by testing the PER was consistent with results obtained in semi-field experiments at colony level^14^.

## Material and Methods

### Bees

Experiments were carried out in May and June 2017 with honeybees (*Apis mellifera carnica*) from a queen-right colony maintained at Würzburg University. Returning foragers were caught individually with glass vials at the entrance of the hive. Bees were regarded as pollen foragers when they had large pollen loads. When they had no pollen loads, they were regarded as nectar foragers. This was most likely because younger bees departing for their orientation flights only leave the hive at noon or in the afternoon^23,24^ and water collectors are a very small subgroup of the foragers^25^.

After individuals were anaesthetized on ice, they were harnessed in holders and fixed with one strip of textile tape between head and thorax and one strip over the abdomen^21^. This way they could freely move their antennae and mouth parts. After ten minutes, bees were fed individually with 5 µl of a 50% sugar solution or of the sugar solution containing one of the following three concentrations of flupyradifurone: 8.3 * 10^−4^ mol/l (1.2 µg ai/bee), 8.3 * 10^−5^ mol/l (120 ng ai/bee), 8.3 * 10^−6^ mol/l (12 ng ai/bee). After one hour in the incubator (temperature 28 °C, relative humidity 70%) the taste of the honeybees was tested. We used flupyradifurone, Pestanal®, analytical standard by Sigma Aldrich (Steinheim, Germany).

### Determining sublethal doses

In a preliminary test with winter honeybees, we could show that a dose of 1,7 * 10^−4^ mol/l, which is twice as much as our highest dose tested, increased mortality significantly 120 min after feeding (χ^2^ = 7.5, P = 0.006) compared to a control (see Supplementary Table T1).

To identify sublethal doses of flupyradifurone under our experimental conditions, we caught and fixed the bees the same way as for the conditioning and fed the same doses of flupyradifurone and a control once (8.3 * 10^−4^ mol/l, 8.3 * 10^−5^ mol/l, 8.3 * 10^−6^ mol/l). Then we released ten individuals per treatment group into cages, fed them with 50% sugar solution from a prepared 5 ml Eppendorf tube ad libitum and held them in an incubator (temperature 28 °C, relative humidity 60%, see Supplementary Figure S1). We checked for dead animals after 72 hours. We utilized age-controlled bees for this experiment and repeated it twice. We did not distinguish between pollen- and nectar foragers here.

### Quantifying taste

To quantify the taste or gustatory responsiveness of each bee, we presented water and a series of sucrose concentrations (0.1%, 0.3%, 1.0%, 3.0%, 10%, 30%) in ascending order to the antennae of each bee using a tooth pick. On each stimulation it was noted if the bee showed a proboscis extension response (PER). The inter-trial interval was two minutes. The sum of the PER after stimulation with the six different sucrose concentrations and water results in the gustatory response score (GRS) of a bee, which is an excellent measure of its gustatory responsiveness^26–28^. Only bees showing a PER after stimulation with 30% sucrose were selected for the classical conditioning assay, because this sucrose concentration served as unconditioned stimulus and reward^21^.

### Classical olfactory conditioning

We used 1-hexanol as conditioned stimulus (CS+) and 1-nonanol as unconditioned stimulus (CS-) (73117 1-hexanol, 74278 1-nonanol, both Sigma Aldrich, Steinheim, Germany)^29^. Five microliters of each odorant were applied on 1 cm^2^ filter paper which was placed in a 20-ml syringe^29^. Test animals were placed in a constant airflow. First, bees were tested for their spontaneous responses to each odorant for eight seconds. The bees showing spontaneous responses were discarded. For the six conditioning trials only the conditioned odorant 1-hexanol was delivered for eight seconds. In the first four seconds, 1-hexanol alone was delivered to both antennae. While the odorant was applied blown to the antennae, the PER was elicited by touching both antennae with 30% sugar solution. When the bee extended its proboscis, it was allowed to lick from the 30% sugar solution for two seconds while the odorant was still applied. Then the odorant was removed, while the bee could lick sugar water for two more seconds. In each training trial it was recorded whether the bee extended its proboscis during the first four seconds of odorant application. The inter-trial interval was five minutes. The sum of the conditioned responses during the trials constitute the acquisition score of a bee^28^. After conditioning, the bees were fed to satiation with 50% sugar solution and placed in an incubator (temperature 28 °C, relative humidity 70%) for testing memory and extinction on the next day.

### Memory test and extinction

On the next morning, the bees were fed with a maximum of five μl or 15 μl, depending on the time of the extinction test, to avoid starvation. The extinction test was conducted 24 hours after conditioning. Bees were placed in a constant airflow and the same odorants were applied as used for training. We applied 10 extinction and discrimination trials without any rewards. Thus each bee could experience five stimulations of each odorant. Odorants were applied in pseudo-randomized order. After each olfactory stimulation the occurrence of the PER was recorded. The maximally five responses to the conditioned odor constitute the extinction score of the bee^28^. Discrimination was defined as the difference between all responses to the conditioned odor and all responses to the alternative odor^28^. After the last trial, the occurrence of the PER after antennal stimulation with 30% sugar solution was tested once. Only data from bees responding to 30% sugar solution were used for analysis.

### Statistics

Statistical analyses were conducted using SPSS Statistics 23 (IBM, Armonk, United States of America). The mortality rates of the differently treated groups were compared using Pearson Chi-square Tests (χ^2^) with Bonferroni correction. Data was tested for normal distribution using a Kolmogorov Smirnov Test. The sucrose-concentration-response curves and the learning curves were compared using Logistic Regression (“lreg”), since data did not follow normal distribution^30^. For post-hoc multiple comparisons we used the Least Significant Difference Test.

The number of bees showing spontaneous responses to the conditioned and the unconditioned odorants were tested using Pearson Chi-Square Test with Bonferroni correction. As gustatory scores, acquisition scores, extinction scores and discrimination scores were not distributed normally, we applied non-parametric analysis of variance (Kruskal-Wallis H Test) to determine the effect of flupyradifurone between the different treatment groups. Dunn’s Post-Hoc-Tests were applied for pairwise comparisons.

### Data availability

All data generated or analyzed during this study are included in this published article and its Supplementary Information files.

## Results

### Survival

We first determined sublethal doses of flupyradifurone in two repetitive experiments. We counted the dead animals in each cage 72 h after initial feeding of flupyradifurone. Chi square Test revealed no significant difference in the number of dead bees between the differently treated groups and the control (First trial: χ^2^ = 4.0, P = 0.265; Second trial: χ^2^ = 2.2, P = 0.528, see Supplementary Table T2).

### Taste behavior

**Figure 1:**
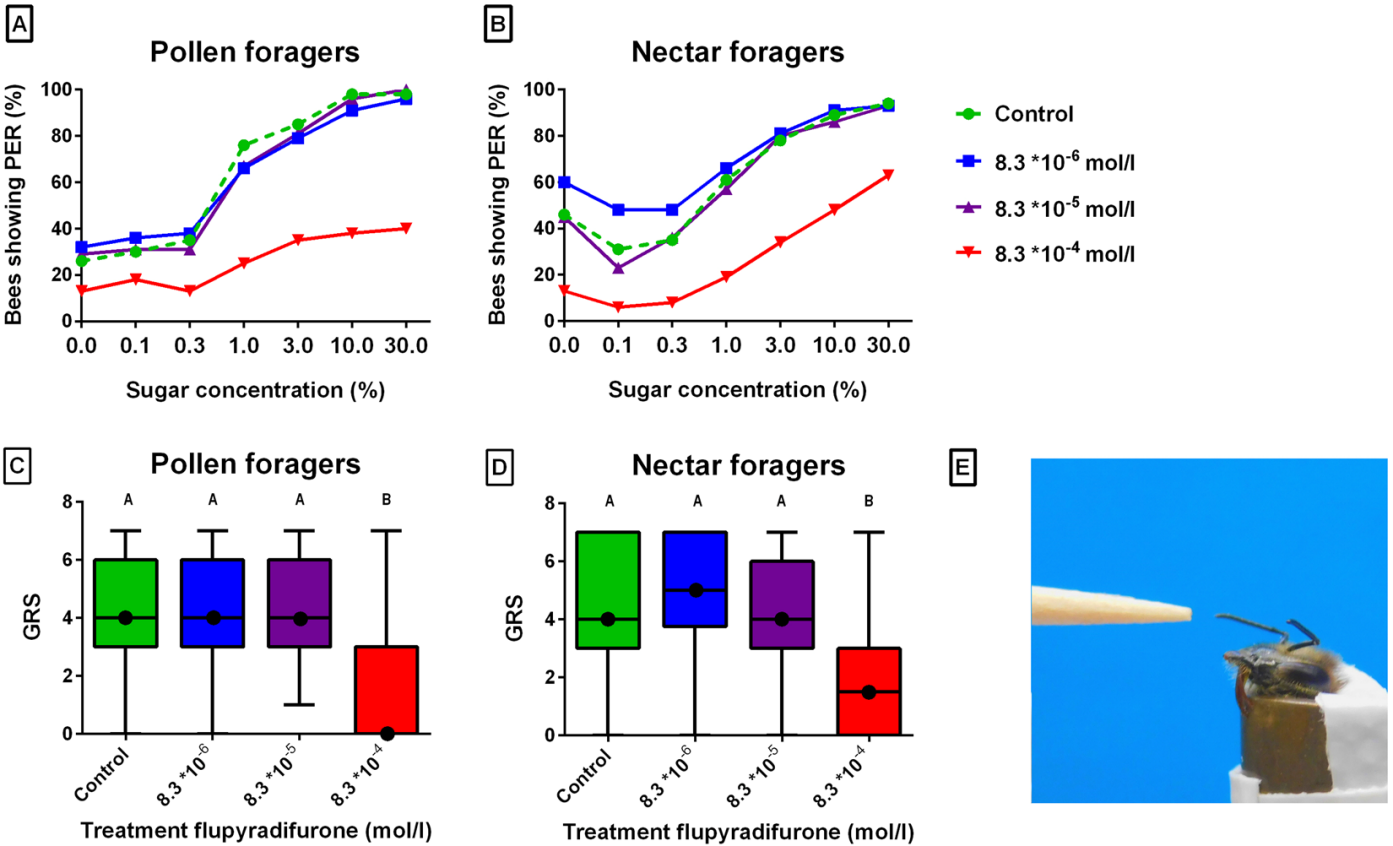
(**A**,**B**) Gustatory response curves of untreated bees (dotted) and flupyradifurone-treated bees in pollen foragers (**A**) and nectar foragers (**B**). Numbers of bees showing PER treated with flupyradifurone in the concentration of 8.3*10^-4^ mol/l were significantly lower than in the controls among the pollen foragers (P < 0.001) and the nectar foragers (P < 0.001). (**C**,**D**) Median gustatory response scores (GRS; intermediate lines) and quartiles (upper and lower lines) of untreated bees and flupyradifurone-treated bees in pollen foragers (**C**) and nectar foragers of the honeybee (**D**). Bees treated with 8.3 *10^-4^ g mol/l flupyradifurone had significantly lower GRS than the controls in the pollen forager group (P < 0.001) and in the nectar forager group (P < 0.001). For the numbers of bees per treatment see Table 1, for the test results see Supplementary Tables T3, 4. (**E**) Fixed bee.

In pollen and nectar foragers, the percentage of bees showing a PER increased with increasing sugar concentrations, indicating a preference for higher sucrose concentrations across treatments. Treatment had a significant effect on the taste-response curves in both groups of foragers (Fig. 1A,B; pollen and nectar foragers: P < 0.001; logr.). Pollen and nectar foragers which were treated with flupyradifurone in the concentration of 8.3*10^-4^ mol/l responded significantly less frequently to the different sucrose concentrations compared to the control groups (Fig. 1A,B; pollen and nectar foragers: P < 0.001).

**Table 1:** Number of bees per treatment tested for their gustatory responsiveness (GRS) and acquisition, extinction and discrimination performance.

	Pollen foragers					Nectar foragers				
	GRS	Spontaneous responses	Acquisition	Extinction	Discrimination	GRS	Spontaneous responses	Acquisition	Extinction	Discrimination
Control	46	45	42	30	23	54	50	38	26	14
8.3 * 10^-6^ mol/l	47	44	39	25	21	58	53	39	21	13
8.3 * 10^-5^ mol/l	48	48	45	32	25	56	50	39	23	15
8.3 * 10^-4^ mol/l	55	22	19	18	6	62	37	36	27	11

The effect of flupyradifurone on gustatory responsiveness is further demonstrated by a significant reduction in GRS (Fig. 1C,D; pollen foragers: KW = 43.0, P < 0.001; nectar foragers: KW = 52.6, P < 0.001). Pollen and nectar foragers treated with flupyradifurone in the concentration of 8.3 *10^-4^ mol/l displayed significantly lower GRS than control bees (pollen foragers P < 0.001; nectar foragers P < 0.001). The two lower concentrations of flupyradifurone did not affect GRS compared to the control (Fig. 1C,D).

These data indicate that flupyradifurone reduced taste for sugar in a dose-dependent manner equally in bees collecting pollen and those collecting nectar.

### Classical olfactory conditioning of the PER

Only bees which responded to 30% sucrose solution were used for conditioning, because this concentration was used as rewarding stimulus (see Material and Methods). Because flupyradifurone in the concentration of 8.3 *10^-4^ mol/l strongly reduced gustatory responses, only few bees of this group could be analyzed for their learning performance.

Of these bees showing a PER towards 30% sugar, among the pollen foragers, the GRS did not differ between the treated and the untreated groups (KW = 0.6, P = 0.902), but in the nectar foragers the GRS of the highest treated group was significantly lower compared to the control (P < 0.01).

For the conditioning, first, the spontaneous responses to the conditioned stimulus 1-hexanol (CS+) and those to the unconditioned stimulus 1-nonaol (CS−) were tested. Bees were regarded as responding spontaneously if they showed the PER either to CS+ or CS−. Among the nectar foragers, Chi Square Test revealed a significant effect of treatment on spontaneous responses to the odorants (χ^2^ = 11.1, P < 0.05). Nevertheless, there was no significant difference between individual treatment groups among the pollen foragers (χ^2^ = 3.0, P = 0.388), suggesting a weak effect of treatment on spontaneous responses.

**Figure 2:**
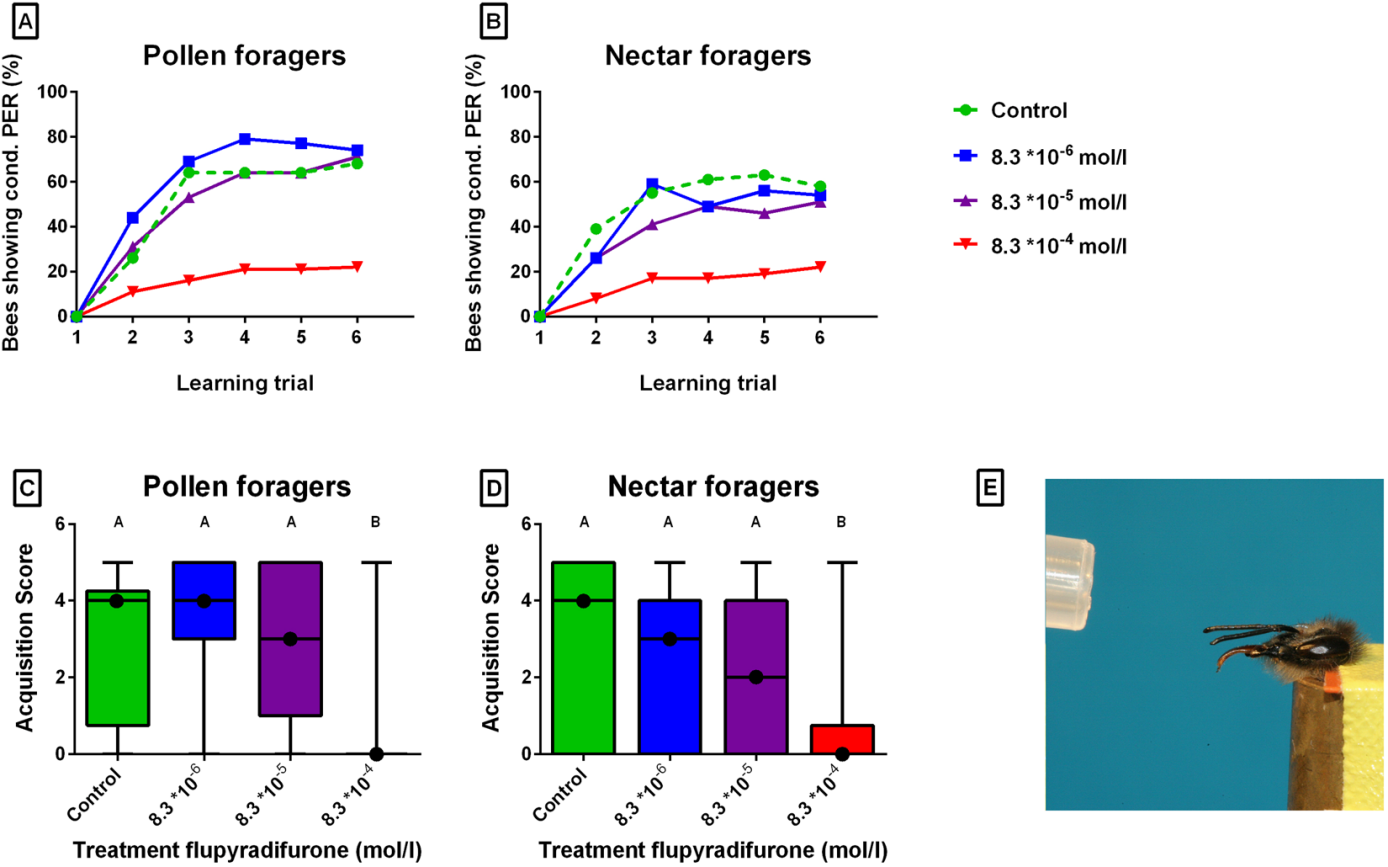
(**A**,**B**) Acquisition curves of untreated bees (dotted) and treated bees in pollen foragers (**A**) and in nectar foragers (**B**). In all groups, the number of bees showing the conditioned proboscis extension response (cond. PER) increased with learning trials The learning curves of bees treated with the flupyradifurone in the concentration of 8.3 * 10^−4^ mol/l differed significantly from the control in pollen foragers (P < 0.001) and nectar foragers (P < 0.001). (**C**,**D**) Median acquisition score (intermediate lines) and quartiles (upper and lower lines) of untreated bees and flupyradifurone-treated bees in pollen foragers (**C**) and in nectar foragers (**D**). There was a significant difference between flupyradifurone in the concentration of 8.3 * 10^−4^ mol/l and the control in pollen foragers (P < 0.005) and in nectar foragers (P < 0.001). For the numbers of bees per treatment see Table 1, for the test results see Supplementary Tables T3, 4. (**E**) Bee showing conditioned PER.

Flupyradifurone significantly inhibited the learning performance of bees (Fig. 2A,B; pollen and nectar foragers: P < 0.001, logr.). In both groups of foragers, the number of bees showing the conditioned proboscis extension response increased with learning trials, demonstrating that an increased number of bees associated the conditioned odor with the reward. Bees treated with flupyradifurone in the concentration of 8.3 *10^-4^ mol/l had a significantly lower learning curve compared to the controls in pollen foragers (P < 0.001) and nectar foragers (P < 0.001).

Similarly, acquisition scores were significantly affected by treatment in pollen foragers (KW = 18.1, P < 0.001) and in nectar foragers (KW = 18.5, P < 0.001). Flupyradifurone in the concentration of 8.3 *10^-4^ mol/l significantly lowered the learning performance both in pollen foragers (P < 0.005) and in nectar foragers (P < 0.001) compared to the control group. The two lower concentrations did not lead to a significantly reduced learning performance (Fig. 2C,D).

### Memory test and extinction

**Figure 3:**
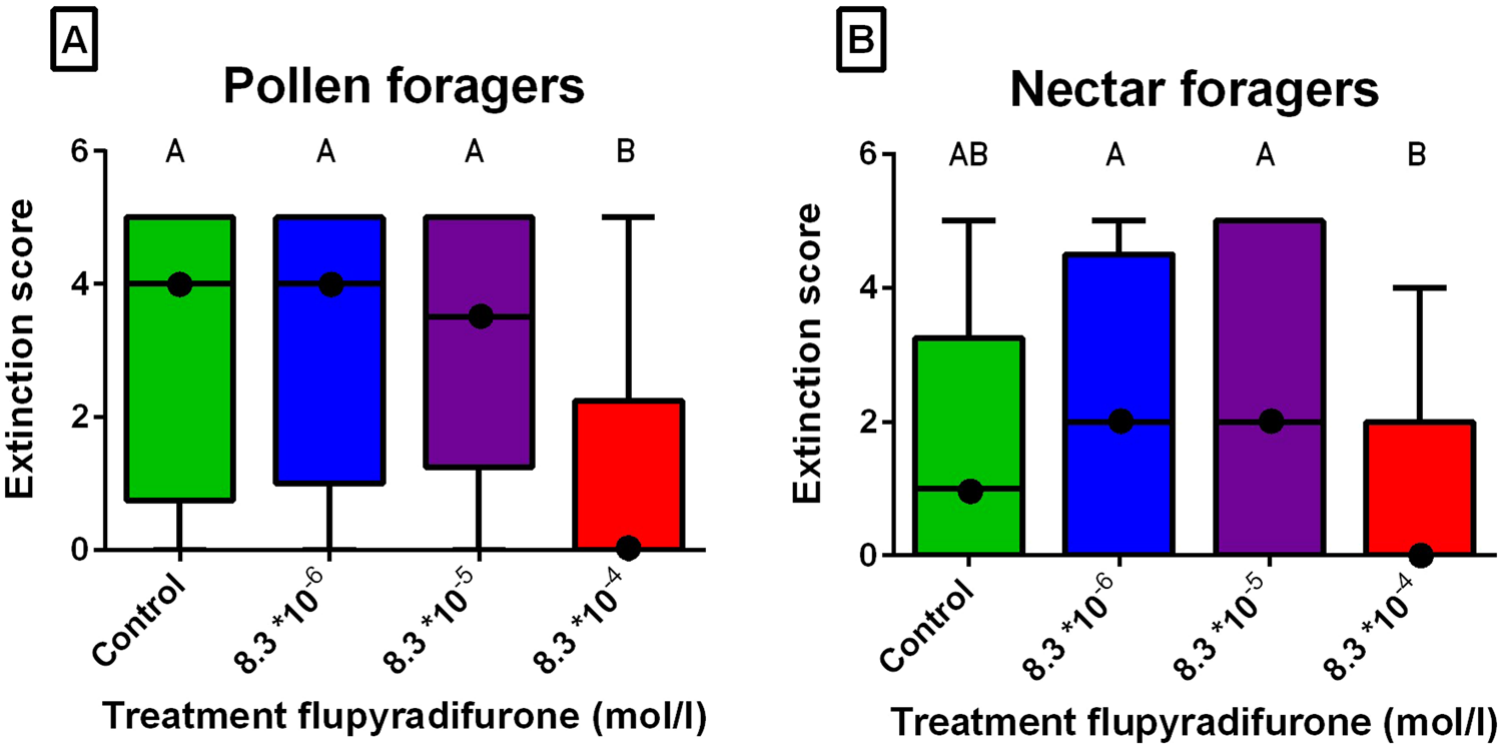
(**A**,**B**) Median extinction score (intermediate lines) and quartiles (upper and lower lines) of untreated bees and flupyradifurone-treated bees in pollen foragers (**C**) and in nectar foragers (**D**). There was a significant difference between flupyradifurone in the concentration of 8.3 * 10^−4^ mol/l and the control in pollen foragers (P < 0.005). In nectar foragers 8.3 * 10^−4^ mol/l was significantly different from 8.3 * 10^−5^ mol/l (P < 0.05) and 8.3 * 10^−6^ mol/l (P < 0.05). For the numbers of bees per treatment see Table 1, for the test results see Supplementary Tables T3, 4.

Extinction scores differed significantly between the differently treated groups and the control group among the pollen and nectar foragers (pollen: KW = 14.6, P < 0.005; nectar: KW = 8.9, P < 0.05). In pollen foragers, flupyradifurone (8.3 *10^-4^ mol/l) significantly lowered the memory performance compared to the control (P < 0.05). In nectar foragers, the same trend was observable, but here this difference was not significant (Fig. 3A,B; P = 0.116).

Nevertheless, we could not find a difference in the discrimination scores, neither in pollen foragers (KW = 0.7, P = 0.869) nor in nectar foragers (KW = 0.8, P = 0.860).

## Discussion

The aim of this study was to determine the effect of the novel pesticide flupyradifurone on the taste for sugar and cognitive abilities of honeybee foragers.

We found no difference in the mortality rate of bees treated with the different concentrations of flupyradifurone and the controls, demonstrating that all doses of this pesticide which we employed were sublethal. Our findings are intriguing, because in some experiments^31^ our highest dose (i.e. 8.3 *10^-4^ mol/l = 1.2 µg ai/bee) was shown to act as LD_50_, although our treated bees survived very well with this treatment.

Nevertheless preliminary data showed a significantly higher mortality rate for the double LD_50_. However, different mortality rates for the same agent are frequently described for other pesticides^32^.

Our data demonstrate that flupyradifurone, though administered in a high concentration (8.3 *10^-4^ mol/l), can significantly reduce gustatory responsiveness and impair associative learning and memory in honeybee foragers, regardless of whether they collect pollen or nectar.

How the pesticide interferes with taste and cognition in honeybees is unclear. Flupyradifurone binds to nAchR with an agonistic efficacy of 0.56 relative to acetylcholine (Ach)^18^. Surprisingly little is known about the molecular properties and cognitive functions of these receptors, even though literature on nAchR sensitivity to insecticides is abundant. Once Ach is released into the synaptic cleft, it quickly binds to its receptors before being hydrolyzed by acetylcholinesterase (AchE). Marking AchE in the brain Kreissl and Bicker^33^ showed by immunohistochemistry that AchE positive sensory fibers of the antennal nerve project into the dorsal lobe and the suboesophageal ganglion. Similarly, AchR-like immunoreactivity was found in the subesophageal ganglion. Both dorsal lobe and subesophageal ganglion process gustatory information from the antennae^34^. In addition, the lips of the mushroom bodies, which receive input from antennal olfactory sensilla, display both AchE-like and AchR-like immunoreactivity. Similarly, it was shown in *Drosophila melanogaster* larvae that AchR subsets of olfactory and gustatory afferents are cholinergic^35^.

These findings suggest a role of Ach in the processing of gustatory and olfactory signals from the antennae in the honeybee brain. Our data directly support this assumed function of Ach, because blocking of AchR resulted in reduced responsiveness to sugar and reduced olfactory learning performance. This effect on gustation and cognition is similar to that induced by the neonicotinoid imidacloprid (for review see refs^6,36^), which binds to the same nAChR. This phenomenon has also been observed in honeybees with reduced responsiveness to sucrose^37^, suggesting an effect of AchR on taste perception or processing. Like our observation in olfactory conditioning, the classical blocker of AchR, mecamylamine, led to a reduced learning performance^6^. These findings suggest that the behavioral impairment we observed after flupyradifurone treatment resulted from an inhibition of nAchR in the honeybee brain.

It was hypothesized that there are α-bungaratoxin (α-BGT) insensitive nicotinic receptors which are essential for retrieval processes and α-BGT sensitive receptors essential for the formation of long-term memory^6^. As we detected an effect on long-term memory, it seems reasonable that both subtypes of nAchR are affected.

An important question is in how far honeybee foragers in the field will be exposed to the dose of our experiment which led to significant behavioral deficits. So far, few studies have investigated the residues of flupyradifurone in nectar and pollen. Campbell *et al*.^38^ applied flupyradifurone on buckwheat fields at the maximum cumulated seasonal application rate allowed by the label (i.e. 410 g ai /ha) per individual foliar application. They found a maximum amount of flupyradifurone of 541 ppb in nectar and 1170 ppb in pollen^38^. Presuming that a honeybee consumes ~5 mg of pollen^39^ and ~30 mg of honey per day^40^ the maximum dose a bee consumes per day is 54 times lower than the dose that significantly reduced taste and learning performance of honeybees in our experiments, 8.3 * 10^−4^ mol/l (1.2 µg ai/bee). That means it would take the bee approximately 54 days to incorporate this amount of flupyradifurone. This is based on the assumption that the active substance is not metabolized and that no detoxification takes place. An actively foraging honeybee usually dies after 3–4 weeks^24^. Therefore it seems unlikely that she would consume this amount of flupyradifurone over the summer season. As some beekeepers leave larger amounts of honey in the hive for overwintering, long-lived winter bees feeding on honey, however, might be exposed to an accumulated amount of flupyradifurone and possibly other pesticides^5^.

Flupyradifurone can not only be applied per individual foliar application but also per soil drench and seed treatment, depending on the target plants. It has a broad spectrum of target plants including vegetables, fruits, grapes, coffee and cocoa, and can be applied during a wide application window^18^. These crops will most likely also be treated with other pesticides what can lead to a multitude of pesticides found in pollen^4^ and honey^5^. Therefore, different pesticides can possibly affect honeybees at the same time. Additive negative effects of pesticides and anti *Varroa* treatments on behavior have been demonstrated for honeybees^11^ and additive effects on toxicity of honeybees of imidacloprid in combination with a few other pesticides were shown^41^. It is therefore reasonable to investigate in future studies the combined effects of flupyradifurone and other stressors on honeybees.

In addition, next to nothing is known about the effect of flupyradifurone on other insect pollinators including wild bees. Because crop pollination is most efficient when wild bees and honeybee forage in the respective fields^42^ the effects of flupyradifurone on wild bees are an important topic.

Our experiments only shed light on one way of treatment. Further studies need to investigate the effect of a long term treatment on honeybee behavior, such as gustatory responsiveness, learning and memory. In addition, we still know nothing about possible effects on dancing or orientation behavior in honeybees.

## Conclusions

Our data show that the novel pesticide flupyradifurone (Sivanto, Bayer AG) can potentially reduce the taste and cognitive skills of honeybee foragers. In our experiments, only the highest concentration had significant effects. Most likely, honeybee foragers will not be exposed to these high concentrations. Therefore, the appropriate use of this pesticide can be considered safe for honeybees, at least with respect to the behaviors studied here and under field conditions when applied according to label instructions as demonstrated by Campbell *et al*.^38^.

Nevertheless flupyradifurone as well as other pesticides most likely will not be applied on its own. Instead, honeybees will be simultaneously exposed to several pesticides in different crops. It has recently been shown that 75% of the honey worldwide contains one or more different neonicotinoid pesticides^5^.

It is therefore reasonable to investigate in future studies effects of flupyradifurone on more complex behaviors such as complex learning tasks or navigation and to investigate the combined effects of flupyradifurone and other pesticides on honeybees.

## Electronic supplementary material

Supplementary information

Supplementary Dataset 1
