# Supplementary material for: Effects of the novel pesticide flupyradifurone (Sivanto) on honeybee taste and cognition
Source: Sci Rep. 2018 Mar 21;8:4954. doi: 10.1038/s41598-018-23200-0 (PMC5862975; doi:10.1038/s41598-018-23200-0)
Supplement: Supplementary file 1 — Supplementary information [file 41598_2018_23200_MOESM1_ESM.pdf]

Supplementary information

Effects of the novel pesticide flupyradifurone (Sivanto) on honeybee taste and cognition

Hannah Hesselbach[1], Ricarda Scheiner[1]*

[1] University of Würzburg, Biocenter, Behavioral Physiology and Sociobiology,
Am Hubland, 97074 Würzburg, Germany

	Test	Value	df	P-value
**Mortality 120 min after 1,7 $*10^{-4}$ mol/l flupyradifurone**	Chi Square Test	7.5	1	0.006

Supplementary table T 1: Test results of preliminary mortality study.

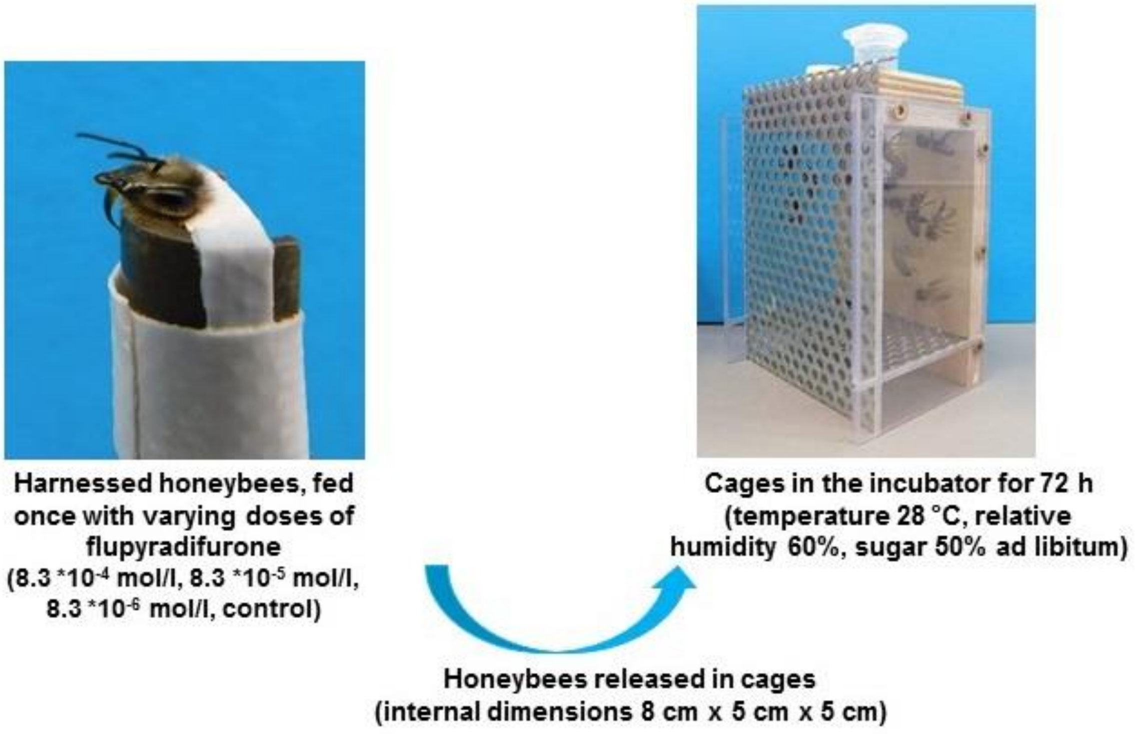

Supplementary Figure S 1: Schematic figure, showing the practical performance of the mortality experiment.

	Test	Value	df	P-value
**First trial**	Chi Square Test	4.0	3	0.265
**Second trial**		2.2	3	0.528

Supplementary table T 2: Test results of the mortality study.

	Treatment	Test	Value	df	P-value
**Taste behavior**		Logistic Regression	51.3	3	.000
	$8.3 * 10^{-6}$ mol/l	Least Significant Difference Test		1	.808
	$8.3 * 10^{-5}$ mol/l			1	.731
	$8.3 * 10^{-4}$ mol/l			1	.000
**Taste behavior**		Kruskal-Wallis H Test	43.0	3	.000
	$8.3 * 10^{-6}$ mol/l	Dunn's Post-Hoc-Test		1	.798
	$8.3 * 10^{-5}$ mol/l			1	.757
	$8.3 * 10^{-4}$ mol/l			1	.000
**GRS[1]**		Kruskal-Wallis H Test	.6	3	.902
**Spontaneous reactions**		Chi Square Test	3.0	3	.388
**Conditioning**		Logistic Regression	26.6	3	.000
	$8.3 * 10^{-6}$ mol/l	Least Significant Difference Test		1	.166
	$8.3 * 10^{-5}$ mol/l			1	.954
	$8.3 * 10^{-4}$ mol/l			1	.000
**Conditioning**		Kruskal-Wallis H Test	18.1	3	.000
	$8.3 * 10^{-6}$ mol/l	Dunn's Post-Hoc-Test		1	.203
	$8.3 * 10^{-5}$ mol/l			1	.945
	$8.3 * 10^{-4}$ mol/l			1	.001
**Extinction**		Kruskal-Wallis H Test	14.6	3	.002
	$8.3 * 10^{-6}$ mol/l	Dunn's Post-Hoc-Test		1	.382
	$8.3 * 10^{-5}$ mol/l			1	.975
	$8.3 * 10^{-4}$ mol/l			1	.003
**Discrimination**		Kruskal-Wallis H Test	.7	3	.869

Supplementary table T 3: Test results of pollen foraging bees. Post hoc comparisons show treatment against control. For number of bees per treatment see table 1. GRS[1]: GRS of conditioned bees.

	Treatment	Test	Value	df	P-value
**Taste behavior**		Logistic Regression	72.0	3	.000
	8.3 * 10^{-6} mol/l	Least Significant Difference Test		1	.200
	8.3 * 10^{-5} mol/l			1	.696
	8.3 * 10^{-4} mol/l			1	.000
**Taste behavior**		Kruskal-Wallis H Test	52.6	3	.000
	8.3 * 10^{-6} mol/l	Dunn's Post-Hoc-Test		1	.242
	8.3 * 10^{-5} mol/l			1	.700
	8.3 * 10^{-4} mol/l			1	.000
**GRS[1]**		Kruskal-Wallis H Test	14.9	3	.002
	8.3 * 10^{-6} mol/l	Dunn's Post-Hoc-Tests		1	.819
	8.3 * 10^{-5} mol/l			1	.299
	8.3 * 10^{-4} mol/l			1	.001
**Spontaneous reactions**		Chi Square Test	11.1	3	.011
**Conditioning**		Logistic Regression	25.2	3	.000
	8.3 * 10^{-6} mol/l	Least Significant Difference Test		1	.491
	8.3 * 10^{-5} mol/l			1	.180
	8.3 * 10^{-4} mol/l			1	.000
**Conditioning**		Kruskal-Wallis H Test	18.5	3	.000
	8.3 * 10^{-6} mol/l	Dunn's Post-Hoc-Test		1	.627
	8.3 * 10^{-5} mol/l			1	.234
	8.3 * 10^{-4} mol/l			1	.000
**Extinction**		Kruskal-Wallis H Test	8.9	3	.031
	8.3 * 10^{-6} mol/l	Dunn's Post-Hoc-Test		1	.299
	8.3 * 10^{-5} mol/l			1	.316
	8.3 * 10^{-4} mol/l			1	.116
**Discrimination**		Kruskal-Wallis H Test	.8	3	.860

Supplementary table T 4: Test results of nectar foraging bees. Post hoc comparisons show treatment against control. For number of bees per treatment see table 1. GRS[1]: GRS of conditioned bees.
